# Evaluation of the Spike Diversity of Seven Hexaploid Wheat Species and an Artificial Amphidiploid Using a Quadrangle Model Obtained from 2D Images

**DOI:** 10.3390/plants13192736

**Published:** 2024-09-30

**Authors:** Evgenii G. Komyshev, Mikhail A. Genaev, Yuliya V. Kruchinina, Vasily S. Koval, Nikolay P. Goncharov, Dmitry A. Afonnikov

**Affiliations:** 1 Institute of Cytology and Genetics, Siberian Branch of the Russian Academy of Sciences, 630090 Novosibirsk, Russia; 2 Kurchatov Genomics Center, Institute of Cytology and Genetics, Siberian Branch of the Russian Academy of Sciences, 630090 Novosibirsk, Russia; 3 Faculty of Natural Sciences, Novosibirsk State University, 630090 Novosibirsk, Russia

**Keywords:** wheat spike, hexaploid wheat, amphidiploid, diversity, spike morphometry, image analysis

## Abstract

The spike shape and morphometric characteristics are among the key characteristics of cultivated cereals, being associated with their productivity. These traits are often used for the plant taxonomy and authenticity of hexaploid wheat species. Manual measurement of spike characteristics is tedious and not precise. Recently, the authors of this study developed a method for wheat spike morphometry utilizing 2D image analysis. Here, this method is applied to study variations in spike size and shape for 190 plants of seven hexaploid (2*n* = 6*x* = 42) species and one artificial amphidiploid of wheat. Five manually estimated spike traits and 26 traits obtained from digital image analysis were analyzed. Image-based traits describe the characteristics of the base, center and apex of the spike and common parameters (circularity, roundness, perimeter, etc.). Estimates of similar traits by manual measurement and image analysis were shown to be highly correlated, suggesting the practical importance of digital spike phenotyping. The utility of spike traits for classification into types (spelt, normal and compact) and species or amphidiploid is shown. It is also demonstrated that the estimates obtained made it possible to identify the spike characteristics differing significantly between species or between accessions within the same species. The present work suggests the usefulness of wheat spike shape analysis using an approach based on characteristics obtained by digital image analysis.

## 1. Introduction

One of the most important crops is wheat (*Triticum* spp.). It accounts for more than one fourth of the world’s total cereal crop production and is the main source of staple foods for more than one fifth of the global population [1,2]. Breeding of new high-yielding varieties and lines of wheat resistant to biotic and abiotic stresses will largely ensure food security for a significant part of the world’s population. Productivity traits in wheat are predominantly related to the spike size and shape, the number of grains per spike and their weight [3,4]. It is these traits which ultimately determine the yield of a wheat plant.

The main characteristics of the wheat spike shape, size and productivity include length, the number of spikelets, the width for the front and profile sides, the presence of awns and their length and the spike density [5,6,7,8,9]. However, these parameters cannot always reflect the peculiarities of the wheat spike shape. Therefore, several classifications have been proposed to describe the spike shape. One approach is based on geometric description and includes the following shape classes [10]:

- Fusiform (the middle part of the spike is the widest, narrowing toward the apex and partially toward the base);
- Elliptical (spikes of an elongated oval shape);
- Prismatic (spikes of nearly equal width along the entire length, with the exception of the apical and basal parts);
- Cone-shaped (spikes narrowing to the apex from the base);
- Square-headed (spikes expanding toward the apex);
- Cylindrical (spikes having the same cross-section radius along the entire length).

A similar classification of spikes by shape (fusiform, oblong, clavate and elliptical) and density (lax, middense, dense) was proposed by Clark et al. for description of the American wheat varieties [11].

The classification of wheat spike shapes into three main types (compact, normal and spelt) was proposed by Dorofeev [12] and has been successfully used to date [13]. The compact type corresponds to a short, dense spike with a reduced number of spikelets; the normal type corresponds to a spike with parallel sides and a relatively short, square apex; and the spelt type is represented by a pyramidal spike with an elongated stem and tenacious glumes. Despite the coarser description of the shape, this classification is more relevant to the mechanisms of genetic control of spike morphology established as a result of molecular studies [14,15,16,17].

Evaluation of the spike shape and size characteristics is the basis for the study of wheat diversity [18] and its classification into species, landraces, varieties and cultivars [13,19]. Such analysis can include hundreds, thousands or tens of thousands of plants [20,21,22,23]. Evaluation of the phenotypic traits of wheat plants in most of these studies was performed manually. The simplest way to assess spike characteristics is visual assessment (comparison with a template by type), measuring the size with hand tools and manually counting the grains in a spike and weighing them. This is a labor-intensive process. It should also be taken into account that the results of such measurements are usually documented manually and may contain errors.

Recently, high-throughput methods based on the analysis of 2D and 3D digital images have been increasingly used for crop phenotyping [24,25,26,27]. They have been applied to determine spike morphology in detail with high accuracy on the basis of 3D model reconstruction [28,29,30,31], to count the number of spikelets in a spike [32,33] and to estimate spike size [34,35] and glume pubescence [36] on the basis of 2D images. These works demonstrate the effectiveness of digital methods for the analysis of wheat spike characteristics.

Previously, the authors of the present study proposed a method for estimating wheat spike size and shape based on the analysis of digital 2D images obtained under laboratory conditions [37]. This approach makes it possible to identify the spike region in an image, separate its body from the awns and estimate the spike length, the area of its projection in an image and the general shape characteristics (roundness, circularity, etc.). The method allows representing the spike contour using a model of two quadrangles with a common base: the axis of the spike. The parameters of the model quantitatively characterize its shape. The motivation of this study was to provide a digital spike phenotyping method for laboratory imaging and image processing, yielding a set of biologically meaningful parameters describing a spike’s shape and size. The present work makes the following contributions based on this method:

- The spike size and shape diversity in seven hexaploid wheat species and one amphidiploid (190 plants) are evaluated using a simplified (symmetrized) quadrangle model;
- Digital estimates of the spike characteristics are in agreement with the manually measured parameters of the same biological meaning;
- Digitally estimated spike characteristics make it possible to classify spikes both by species and by type with high accuracy through linear discriminant analysis (LDA), where the classification performance increases when manually estimated spike parameters are added;
- This method makes it possible to identify characteristics whose values differ not only between representatives of different species but also between different accessions of the same species.

## 2. Results

### 2.1. Spike Traits and Correlations between Them

First, a test for normality of the distribution of spike characteristics in a sample was performed. The results are shown in Supplementary File S1. For the full set of plants, only four traits followed the Gaussian distribution according to the Shapiro–Wilk test: the area of the quadrilateral in the spike model (q_S), the perimeter of the spike contour in the image (c_P), the spike contour area (c_SA) and the solidity for the spike contour (c_So). The high proportion of traits with distributions differing from the norm can be explained by the fact that the whole sample was characterized by a mixture of several distributions corresponding to individual species and accessions. For each individual species or accession, deviations from the normal distribution showed a significantly smaller number of characteristics, especially if the species was represented by a single accession. For example, *Amphiploid speltiforme* (ASP) was represented by one accession and demonstrated reliable deviations from the normal distribution for nine traits. In *Triticum macha* (two accessions) and *Trtiticum yuannanese* (one accession), five and seven traits deviated from the normal distribution, respectively. First of all, these traits included the parameters of the basal and apical parts of the spike, derived traits (normalized to the length) and rugosity (c_Ru), which characterized the irregularity of the spike body border in the image. Parameters such as the length, spike area, size of the central part and similar parameters generally followed a normal distribution for a one- or two-accession sample.

The statistical relationship between the used spike parameters in the examined samples was evaluated (5 manually estimated parameters and 26 parameters estimated from image analysis). The results are summarized in Figure S1 (Supplementary File S2). The figure shows the presence of a large number of pairs of traits whose values were closely related.

**Figure 1:**
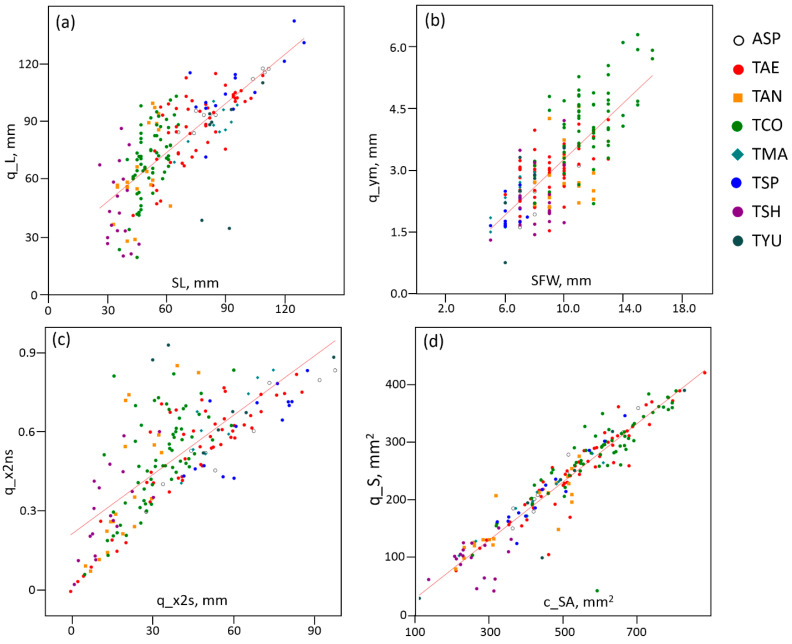
Scatter plots for some pairs of traits with high values for correlation coefficients: (**a**) SL and q_L; (**b**) SFW and q_ym; (**c**) q_x2s and q_x2ns and (**d**) c_SA and q_S. Units are given next to the axis names, and the parameter q_x2ns is dimensionless. Red lines show the straight line of regression. The correspondence of the marker shape and color to the wheat species or amphidiploid is shown in the top right.

First of all, the relationship between manually measured and image-derived traits should be considered. The strongest relationship was observed between the spike length (SL) and spike model parameters such as the length estimate (q_L; Figure 1a) and central segment length (q_x2s). For these trait pairs, the correlation coefficients were the highest (0.76). The spike contour perimeter (c_P) had the second highest correlation coefficient with the SL parameter (0.59). The spike length (SL) was negatively correlated with the spike density index (SDI) (*r* = −0.78), and accordingly, this parameter had negative correlation coefficients with spike characteristics obtained from the image, namely q_L, q_x2s and c_P (−0.61, −0.68 and −0.51, respectively).

The spike frontal width (SFW) showed high values for its correlation coefficients with the traits characterizing the spike width in the image q_ym (0.76; Figure 1b) and y_2s (0.56). A high value for the correlation coefficient was also observed between the SFW and the traits of the spike area, namely the quadrangle area (q_S) and spike contour area (c_SA). Since the side width (SSW) showed a positive relationship with the width of frontal projection, the above mentioned traits (q_ym, y_2s, q_S and q_SA) showed high correlation coefficients with this trait as well. Thus, the obtained results demonstrated that the estimates of such spike parameters as the length and width, obtained manually and based on the analysis of digital images, showed a significant statistical relationship.

The statistical relationship between numerical traits in most cases reflected the fact that some traits were derived from others. The former included normalized traits, such as the length of the central segment (q_x2s) and the same value normalized to the spike length (q_x2ns; Figure 1c). High values for the correlation coefficients were also observed between the spike size and area characteristics in the quadrangle model (q_L and q_S, q_x2s and q_S2, etc.). Significant correlations were also found between different estimates of the same spike characteristic, such as the area (q_S and c_SA; Figure 1d) or shape (c_CI and c_R).

It should be noted that the absolute values of the traits also agreed well. For example, the model parameter q_ym can be interpreted as an estimate of half of the spike width. (It is the ratio of the quadrangle area to the length of the spike.) In this case, a linear regression of the spike width SFW and quadrangle width q_ym (Figure 1b) yielded a slope estimate of 0.38 (close to 0.5) and a bias of −0.12 (close to 0). The parameter q_S characterized the area of the quadrangle and therefore estimated half the area of the spike. A linear regression of q_S and c_SA (Figure 1d) yielded a slope estimate of 0.5. The intercept value of −21.8, however, indicates a bias for these two parameters, where q_S was systematically smaller due to the fact that the edges of the quadrangle approximated the contour of the spike. For the parameters SL (x) and q_L (y), the regression yielded estimates of 0.8 (close to 1) for the slope and 22.8 for the intercept. This also indicates a systematic bias; the estimates obtained by image analysis were ~2–3 cm higher compared with the manual estimate of the spike length. This may be a consequence of the fact that the manual measurement identified the positions of the spike apex and base by eye, while the computer analysis identified these positions as the outermost pixels of the contour.

**Figure 2:**
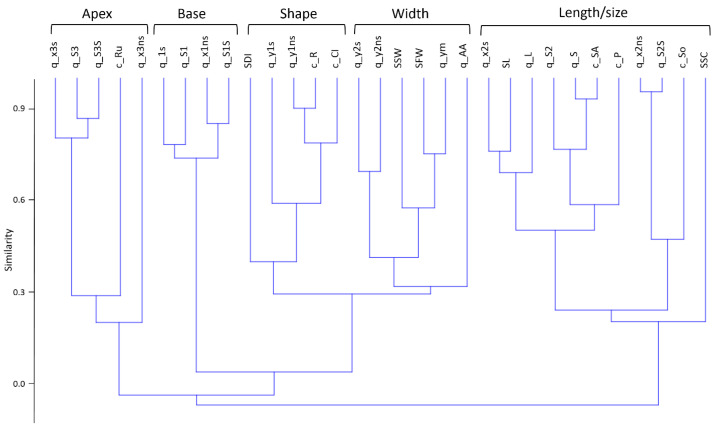
Tree diagram of the similarity of 31 wheat spike traits based on their Pearson correlation coefficients in a sample of 190 plants. Brackets above the tree show five main clusters, and their generalized classifications are given. The similarity axis of the traits is given on the left.

The similarity of the features to each other, evaluated on the basis of the Pearson correlation coefficients and hierarchical clustering by the unweighted pair group method with an arithmetic mean, is demonstrated in the form of a tree in the diagram in Figure 2.

Five large clusters can be identified in the resulting tree (Figure 2). The first cluster (from left to right) includes the characteristics of the spike apex size and area (third segment) as well as the spike contour rugosity (c_Ru). The second cluster includes characteristics of the basal part of the spike (first segment). The third cluster includes the general shape characteristics: roundness (c_R), circularity (c_CI), density index (SDI) and spike base width (q_y1s). The fourth cluster includes traits related to the spike width. The fifth cluster includes traits related to the spike length and size (SL, q_L, c_SA and c_P), characteristics of the central (second) segment (q_x2s, q_S2, q_x2ns and q_S2S) and, interestingly, the number of manually counted spikelets (SSC).

Thus, in the proposed model, the spike shape was characterized by three main segments: the base, central part and apex. Within each segment, the parameters showed high statistical dependence. The clusters of these parameters may reflect both the peculiarities of genetic control of the spike shape and the specificity of the model for describing its shape. The other two clusters reflect the general shape parameters (size, elongation, roundness and area of awns, etc.).

### 2.2. Diversity of Wheat Spikes in Size and Shape

The mean values and standard deviations of some basic characteristics were estimated for each of the studied species and for the amphidiploid. The obtained estimates for the whole sample and individual species as well as the amphidiploid are summarized in Table 1.

Table 1 shows the wide variation in spike traits among the species. For example, the manually estimated spike length (SL) ranged from a minimum of 35 mm in *T. sphaerococcum* (TSH) to a maximum of 94 mm in *T. spelta* (TSP). Parameters such as the quadrangle length q_L, central segment length q_x2s and roundness c_R were also extreme in these species. The spike width SFW and its digital estimate q_ym were maximal in *T. compactum* and minimal in *T. macha* (TMA) and *T. spelta* (TSP). These extreme differences were also observed in these species for the parameter c_Ci. The spike areas in the model (q_S) and for the spike contour (c_SA) were maximal in *T. compactum* (TCO) and minimal in *T. sphaerococcum* (TSH).

In general, *T. spelta*, *T. compactum* and *T. sphaerococcum* can be referred to as the most extreme species in terms of spike characteristics. The first one demonstrated the most elongated loose spike, the second one was characterized by the most rounded and compact spike, and the third one had the spike of the smallest size. The smallest number of parameter values reaching the extremum in the considered sample was observed in *T. aestivum* (TAE), *T. antiquorum* (TAN), *T. macha* (TMA) and *Amphiploid speltiforme* (ASP).

Regarding awns, the maximum value of the awn area was observed in *T. compactum*, and the minimum value was observed in the amphidiploid.

**Figure 3:**
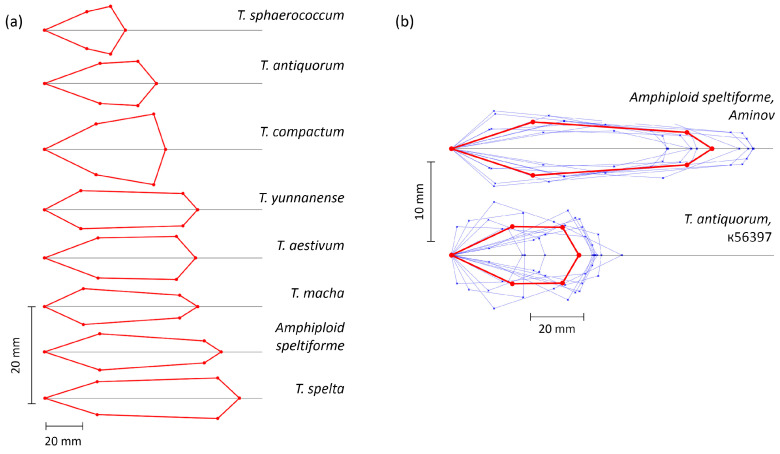
Visualization of spike quadrangle models. (**a**) Models derived from mean parameter values for 7 wheat species and the amphidiploid. (**b**) Models derived for individual spikes of *Amphiploid speltiforme* and sample k-56397 of *T. antiquorum* (thin blue lines) as well as for their mean values (red lines). Vertical and horizontal size scales are shown next to the diagrams. The vertical scale was multiplied to visualize the spike shape more clearly.

A visual graphical representation of the averaged parameters of the spike models for seven species and the amphidiploid is presented in Figure 3a. This visualization clearly shows not only differences in the length and size of the spikes but also in the shape of their segments. Thus, in *T. compactum* and *T. sphaerococcum* the apical segments were broader than the basal ones, while in *Amphiploid speltiforme* and *T. macha*, the opposite was true. In *T. yuannanese* and *T. aestivum*, the sizes of the apical and basal segments were approximately the same.

Figure 3b clearly demonstrates the diversity of the spike shapes in individual plants of *Amphiploid speltiforme* and *T. antiquorum* accession k-56397. This demonstrates that for different plants of the same genotype, there was diversity in both the length and width of the spikes and the shape of their various segments.

In the spelt spikes, differences in the spike length (the longest one was found for *T. spelta*) were observed, but at the same time, the ratios of the spike widths at the base and apex differed among them. For example, in *T. spelta*, the base was narrower. For normal spikes (*T. aestivum* and *T. yuannanense*), their shape and size were similar, but in *T. yuannanense*, the spike at the base was wider than that at the apex. The compact spikes (*T. sphaerococcum*, *T. antiquorum* and *T. compactum*) were club-shaped but varied greatly in length and width. In addition, in *T. antiquorum*, the lateral sides of the spike were practically parallel, unlike the spikes of the other two species.

Analysis of variance (ANOVA) was performed for the relationship between the species and spike characteristics. It revealed a number of parameters statistically significantly different when comparing the mean values in spikes of different species. These results were also confirmed using Levene’s and Kruskal–Wallis tests (Table 2).

Table 2 demonstrates that most of the mean values of the spike shape parameters differed significantly among the wheat species. The exceptions were the characteristics of the base and apex lengths and contour rugosity (q_x1s, q_x3s, q_S3 and c_Ru). Interestingly, Table 2 demonstrates concordance in the statistical tests between the spike traits measured manually and digitally (SL and q_L; SFW and q_y). Thus, the quadrangle model made it possible to identify spike traits for which reliable differences between different species were observed.

Similar ANOVA and other tests were performed to compare the mean values of the parameters in plants with different spike types: compact, normal and speltoid. The results are summarized in Table S1 (Supplementary File S2). In this case, it can be seen that in addition to the characteristics of the base and apex, the traits with no significant differences between spikes of different types were related to the area (q_S, q_S2 and c_SA). At the same time, the mean values of the length, width, roundness and circularity were significantly different.

### 2.3. Linear Discriminant Analysis of Spikes Based on Shape Characteristics

The results obtained above demonstrate that most of the spike size and shape parameters were significantly different among different species and spike types. The feasibility of using traits measured manually and digitally for plant classification into both species and types based on LDA was evaluated.

The analysis was performed for the trait sets estimated manually and digitally, both individually and jointly. Additionally, taking into account that not all traits had a normal distribution, they were regularized using the Box–Cox method, and LDA was performed for the data modified in this way. Two types of classification were performed: (1) into species and amphidiploid and (2) by spike shape type. As a measure of accuracy, the proportion of spikes classified correctly was evaluated. The results are summarized in Table 3. Confusion matrices are presented in Tables S2 and S3 (Supplementary File S2).

The performance of the classification into species by traits obtained digitally was greater than that with manually estimated traits (~10% difference). However, a combination of both sets of traits improved performance by another ~10% (up to 87.9%). Additionally, the Box–Cox transform systematically improved the classification accuracy for all trait sets by 1–3% on average.

The results of classifying spikes by shape type showed that the manually estimated traits yielded better performance in comparison with the digitally estimated traits alone. However, combining two groups of traits again improved the classification performance (by ~3%). The Box–Cox transform systematically improved the classification performance for all trait sets by 1–3% on average as well.

Thus, the use of digitally estimated spike characteristics made it possible to effectively classify them into species and by spike type with high accuracy. Combining sets of traits estimated digitally and manually increased classification performance, as did the use of data regularization.

**Figure 4:**
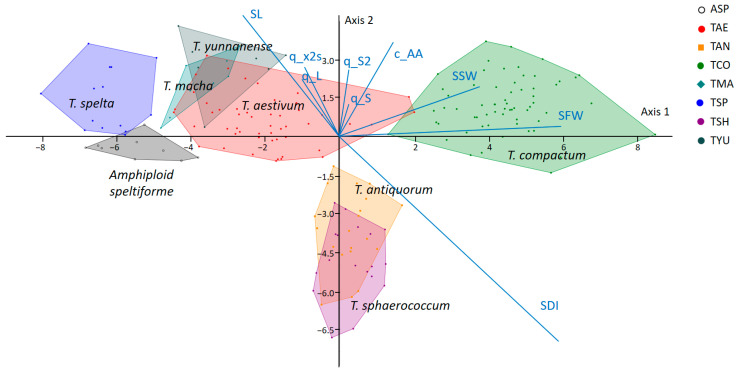
LDA biplot for classification of wheat plants into species using a combined set of species and Box–Cox transformations for input data. The marker types and colors for species and hybrid are shown to the right of the diagram. Polygons for 8 classes are shown by the same color shades as the markers. Blue lines and the traits’ short names show projections for the most important traits in the diagram.

A visualization of the results of the LDA spike classification into species is shown in Figure 4.

Figure 4 shows that such species as *T. spelta*, *T. compactum* and *Amphiploid speltiforme* were separated quite well. In this diagram, overlapping polygons can be observed between the spikes of *T. aestivum*, *T. macha* and *T. yannanense* and between *T. antiquorum* and *T. sphaerococcum*. This agrees well with the model representations in Figure 3; the spike shapes of species with overlapping polygons were quite similar.

As for the traits which contribute to interspecific classification, the greatest variation was observed for such characteristics as the spike length (SL) and spike density (SDI). They were aligned with the line along which *T. spelta* was located on one side and *T. compactum* on the other (Figure 4). Spikes of these species also had the most extreme values for their traits (Table 1). It is interesting to note the division of compact spikes into clusters of *T. compactum* and *T. antiquorum* or *T. sphaerococcum*. They were separated along a line which was almost parallel to the projection of trait of the awn area c_AA. This demonstrates the contribution of the presence of awns to the differences between spikes with compact shapes.

The confusion matrix (Table S3, Supplementary File S2) also shows that the highest number of misclassifications occurred between species whose spikes were similar in shape, and their polygons overlapped in Figure 4.

The diagram of plant classification by spike type is shown in Figure S2 of Supplementary File S2. The figure shows that the plants with compact spikes formed an area which weakly overlapped with the polygons for normal and spelt spikes. At the same time, the greatest overlap was observed for the last two types of spikes. This is consistent with the confusion matrix for classification by type (Table S3, Supplementary File S2). The highest number of misclassified spikes was observed between the spelt and normal classes.

The diagram of plant classification by spike type (Figure S2, Supplementary File S2) also shows that, for the classification by species, the most significant contribution was made by the spike length, width and density index parameters.

### 2.4. Comparison of the Spike Characteristics of Different Accessions of the Same Species

**Figure 5:**
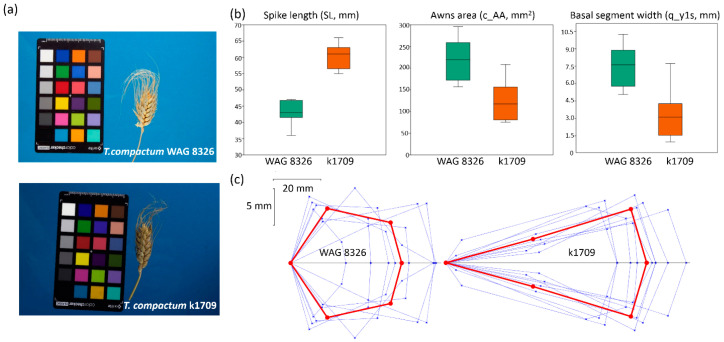
Differences in the morphometric characteristics of the spike in *T. compactum* accessions WAG 8326 and k1709. (**a**) Examples of images of two spikes at the same scale. (**b**) Box and whisker plots for the distribution of spike traits in two accessions (spike length, awn area and basal segment width). (**c**) Visual representation of the spike quadrangle models for individual WAG 8326 and k1709 spikes (thin blue lines) and average model parameters (red lines). Vertical and horizontal scales are shown in the upper left part of the panel.

Regarding the ability of our method to identify differences in the characteristics of spikes from different accessions of the same species, spikes of *T. compactum* and accessions WAG 8326 and k1709 were analyzed. Example images of these spikes are shown in Figure 5a. The figure shows that there were some notable differences between the spikes. The spikes of WAG 8326 were shorter and had longer awns compared with those of k1709. Manual estimates of the spike length demonstrated significant differences between the two accessions (Figure 5b). However, the use of the quadrangle model made it possible to identify more detailed differences between these accessions. They had a significant difference in awn area in the image (c_AA, Figure 5b). This is consistent with the visual assessment. However, the spikes also differed in shape. Significant differences were observed in the width of the basal segment (q_y1s, Figure 5b) and a number of other characteristics measured either manually or digitally (Table 4).

According to the *t*-test, significant differences in the means were observed for 13 traits out of 21. These were, first of all, the characteristics of the spike length (manual estimate SL, digital estimate q_L and perimeter c_P), shape (density index SDI, circularity c_Ci and roundness c_R), basal segment parameters (q_x1s and q_y1s), width (SWF and q_ym), area (q_S) and awn area (c_AA). Note that in addition to 3 of the 5 characteristics measured manually, 10 of the 16 characteristics estimated digitally demonstrated significant differences for two accessions. Most of them were biologically highly relevant but hard or even impossible to estimate with the manual approach: the spike area (q_S, c_SA), circularity (c_Ci), roundness (c_R) and segment parameters (q_x1s, q_x2s and q_y1s).

The model features are visualized in Figure 5c. The diagram demonstrates that the spikes of WAG 8326 were shorter and had a wider base. As for the apical segment, its shape and size were about the same in the two accessions. Thus, the quadrangle model representation of the spike shape can be useful in distinguishing the differing traits in two accessions of the same species.

## 3. Discussion

Digital morphometry of plants has recently been actively developed and used to solve the problems of studying the biological diversity, classification and taxonomy of plants and the search for genes controlling the size and shape of various plant organs [38,39,40,41,42]. To characterize the complex shapes of objects, methods based on landmarks [43], quantitative indices [44], numerical description of contour curves parametrically [45] and nonparametrically [46] and elliptic Fourier descriptors [47] are used.

Recently, the authors of this study proposed a method of parametric description of the spike contour line with a geometric model using digital images [37]. This approach is based on image processing techniques [38]. It provides a number of digital descriptors for the ear size and shape, including those using a geometric model. These parameters can be used to solve spike analysis problems in the “shallow learning” approach [48] (see Figure 4 and [34,37,49]). Recently, however, deep learning methods based on a multilayer neural network architecture have become increasingly popular in analyzing plant images [48,50,51]. They allow automatic extraction of image characteristics for image segmentation and classification and object detection. The accuracy of these methods is quite high. However, as a rule, the obtained digital characteristics (feature vectors) cannot be interpreted from the point of view of biology. Moreover, they are hidden inside the network architecture. On the other hand, it is important for a biologist to have quantitative descriptions of plant features which complement classical ones. These can be used for a more detailed characterization of plants in collections. These descriptors aim to digitalize traits and thus quantify them into understandable, determinable and measurable attributes convergent to genomics [52]. With this in mind, the proposed method [37] is plausible. It yielded a visually relevant spike model (Figure 3) which was fairly easy to interpret (Figure 2). This may be important for finding genes which control the spike shape and size traits in the future.

In terms of the phenotyping problem, the previously developed spike phenotyping method [37] can be classified as using a laboratory platform based on a digital RGB camera and machine learning algorithms [24]. It does not allow the assessment of the physiological state of a plant, cannot be applied to field studies and is not fully automated or robotized. Its main field of application is the study of large collections of plants and their systematic characterization [52,53]. This area of phenotyping has become increasingly relevant recently [54,55,56,57]. The aim is large-scale germplasm phenotypic characterization. These data, in association with the genotyping information, will be a primary component for all crop-breeding programs [58].

Here, the spike characteristics for seven different hexaploid wheat species were compared using this method. The obtained results demonstrated the high accuracy of the method. Digital estimates of the spike length and width based on this method were consistent with those made manually.

The high accuracy of LDA classification by wheat species and by spike type indicates the usefulness of the proposed numerical metrics. For the classification of spikes into three types, the manually derived features yielded ~5% higher accuracy compared with the numerical ones. However, for the classification into types (eight classes), the method based on numerical features outperformed it. In any case, using the two types of features jointly made it possible to achieve higher classification performance. This is consistent with the conclusions from the work by Conejo-Rodriguez et al., in which it was shown that the combination of a plant’s digital and classical descriptors increased the accuracy of the classification of the *Phaseolus* and *Arachis* genbank accessions [52].

Note that the accuracy obtained in this study is comparable—and in some cases exceeded—the accuracy of solving similar problems using machine learning methods. Earlier, the authors of the study used geometric model features and a random forest algorithm to predict the spike type and obtained a proportion of correctly predicted types of 83.73% (314 out of 375), which is close to the results of the present work (83.16% with digitally estimated parameters; Table 3). Bi et al. [34] used eight spike characteristics estimated from 2D images and a neural network to classify 240 images into four wheat varieties. Their method yielded 88% correctly identified spikes. This is higher than using LDA and 26 spike parameters to classify them into eight species in this work (78% with digitally estimated parameters only; Table 3). However, the combined set of manually and digitally estimated traits and regularization of the data led to a close result (88.42%).

Spike classification into five classes by shape using the length of three segments estimated from digital images was performed by Bi et al. [49], yielding a precision of 93%. In the present work, spikes were classified by shape type into three classes, and the best performance result was slightly above 93% (Table 3).

In the analysis, the characteristics of the spikes for a single projection image were used (“table” protocol). The previous results demonstrated that using multiple projections of the spike (“pin” and “table” protocols) improved their classification accuracy [36,37]. The LDA results indicate, however, that estimates of spike characteristics from only one projection are sufficient for practical purposes while being less labor-intensive for phenotyping.

The results show that the proposed model can be used to detect detailed differences in the spike shape between species as well as between accessions of the same species. Moreover, it proves to be functional at a more general level of shape description and separates the spikes of different species well into compact, normal and spelt spikes.

The proposed approach [37], however, has some limitations. First, there is a systematic bias in the digital estimates of some parameters. For example, the spike length estimates obtained using image analysis were higher than those obtained by manual measurement. Similar trends were observed in the work of Bi et al. [59]. For parameters such as the spike length, this can be explained by differences in which points on the spike were used for estimation when ruler and image analysis were used. Nevertheless, it should be taken into account that the proposed method did not provide estimates which were exactly the same as the expert estimates. Some differences may arise from errors in image segmentation and spike contour identification and, to some extent, from digital straightening of the bent spikes.

The second limitation is that the proposed method does not estimate such an important trait for the breeder as the number of spikelets per spike, unlike approaches specifically developed for this task [32,33]. However, the authors of this study have previously shown that by using spike model parameters and machine learning, it is possible to predict its density [37]. Similarly, Li et al. demonstrated that spike parameters such as the length and area in an image could be used to estimate the number of grains [35].

The third limitation is that it cannot describe correctly the shape of branching spikes, the analysis of which may be of interest in studying the processes of spike development [60,61].

At present, the hierarchical subordination of wheat species is artificial and quite subjective [13,62,63,64]. At the same time, the introduction of modern experimental methods in wheat systematics, including genetic, molecular-biological and digital image analysis, will make it possible to construct a real phylogeny of the genus.

In the future, the proposed method and similar approaches can be used for a wide class of tasks for describing the morphology of wheat spikes in genetic collections, in the study of wheat diversity and in breeding and genetic research.

## 4. Materials and Methods

### 4.1. Plant Material

Plants from the collection of Dr. N.P. Goncharov were analyzed. The sample included plants of seven species of hexaploid wheat and one amphidiploid. Plants were grown in a hydroponic greenhouse under individual isolation and standard conditions of humidity, temperature and light for several seasons. Spikes were annotated manually by Fu Hao and S.R. Tumanyan. The spike structure (length, front and side width, density index and spikelet number) was analyzed according to standard methods.

The list of species examined, along with the number of accessions and plants, is given in Table 5. More detailed information on the accessions and the number of plants is given in Table S4 in Supplementary File S1. In total, the sample included 19 accessions and 190 plants.

A brief characterization of the species studied is given below.

*T. aestivum* is a bread wheat characterized by dense, narrow spikes with a brittle rachis and long awns. The spikes are usually five-flowered and of a normal type. Bread wheat is the leading food crop in a large number of countries in the world. It is the most widespread crop throughout the globe, is highly plastic and includes more than 100 varieties. The spikes are awned with short awns of a uniform length all over or only on the upper spikelets; few forms are awnless. Spikes are determinate, square-headed in cross-sections, dense or lax and 4–18 cm long (excluding awns) [13].

*T. spelta* and its subspecies belong to the so-called spelt wheat, a group of species with filmy grain and brittle spikes [65]. It has been cultivated since the 5th millennium BC. The spikes are relatively long (10–15 cm), lax, straight or slightly curved, white, red, gray-blue or blue-black and determinate, awned or awnless [13].

*T. macha*, a hulled hexaploid wheat, is endemic to the Caucasus area. In Georgia, it has stable yields in different climatic conditions and high resistance to various diseases [66]. It has a filmy spike which is more compact in shape than bread wheat but less compact than that of *T. compactum* [12].

*T. antiquorum* is distinguished from other species on the basis of spike compactness, a rounded grain shape and its winter and spring lifestyle [67]. It is believed to be one of the first hexaploid wheat species cultivated by humans [68,69].

*T. compactum* is a wheat species adapted to low-moisture growing conditions. *T. compactum* resembles bread wheat (*T. aestivum*), and thus it is often considered a subspecies of *T. aestivum* ssp. *compactum*. It can be distinguished by its more compact spike due to its shorter stem segments, which gave it its common name [18]. Kihara suggested that bread wheat originated from a cross between *T. spelta* and *T. compactum* [70]. However, it is now believed that *T. compactum* is a younger species [71]. In addition, the authors of this study have shown that the genes determining the spike shape of *T. compactum* and *T. sphaerococcum* are non-allelic [69].

*T. sphaerococcum* Perc. is sphaerococcum wheat, a narrowly endemic species of hexaploid wheat formerly distributed in northwestern India. It is a spring crop adapted to a dry climate under irrigation conditions [13]. It has attracted the attention of breeders due to a complex of valuable traits: spherical grain shape, resistance to lodging, heat resistance, non-sprouting and high baking qualities.

*T. yunnanense* was discovered in 1938 in the Yunnan province in China and named by Shanbao King in 1959 [72]. It is endemic and still continues to be grown in remote and hilly areas. Yunnan wheat is a primitive hexaploid, but it differs considerably from *T. spelta* and *T. macha*. Yunnan wheat has a spindle-shaped spike, the double-rowed side is slightly wider than the single-rowed side, and the spike is covered with wax. The spike is 9–12 cm long and can reach 16 cm, and the number of spikelets in the spike varies from 16 to 29. The average number of grains per spike is between 40 and 60 or is rarely near 80. Incredibly stiff and fragile spike glumes cover the spikelets tightly, and the grains are difficult to thresh [73].

*Amphiploid speltiforme* is an amphidiploid of *T. dicoccoides* × *Ae. Speltoides* which differs from *T. spelta* and from *T. aestivum* in its spike shape. The participation of *T. dicoccoides* in the formation of hexaploid wheat species is also doubtful, according to the results of its karyotype study. According to Badaeva [71], the species carries a number of rearrangements which are not characteristic of other polyploid wheat species. In addition, it has a different form of genetic control of the spike spelt phenotype [74].

Thus, the studied sample represents a wide genetic diversity, diversity in origin and growth habits as well as diversity in shape, spike size and the presence of awns.

### 4.2. Spike Imaging

Spike images were obtained under laboratory conditions using a “table” protocol as described in previous works [37,75]. The spike was placed onto a transparent sample stage on a table with a blue background. The camera was mounted above the transparent sample stage on a tripod with a boom arm. Two pulsed light sources—Falcon DE-300 (flash intensity of 1.0 and 1.4) and Falcon 60 × 60 soft boxes as light modifiers—and a Canon 600D digital camera with an EF-S 28–135 mm f/3.5–5.6 lens were used. The shooting settings were a shutter speed of 1/160; aperture of 10; ISO of 200 and focal lens of 112 mm in RAW format. The white balance was set according to the ColorChecker white background when developing a RAW file. The distance from the camera to the object was 70 cm, while that from the light sources to the object was 60 cm, the table height was 60 cm, and the height of the transparent sample stage (the distance from the object to the blue background) was 20 cm. One image per spike using this protocol was obtained.

**Figure 6:**
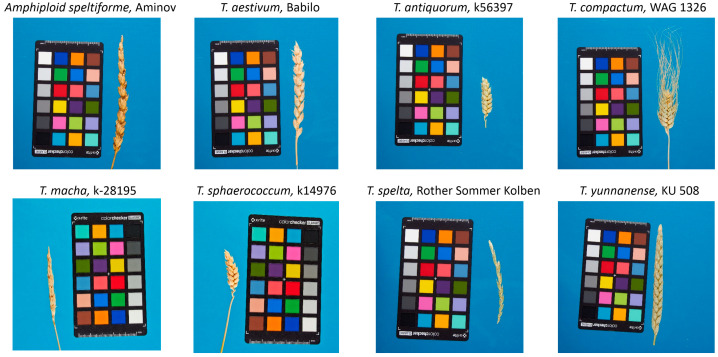
Examples of spike images for 7 wheat species and 1 amphidiploid studied in this work. The names of the species and amphidiploid are given above the images, and the name of the sample or variety is separated by a comma.

Examples of spike images for seven wheat species and one amphidiploid are shown in Figure 6. The spikes are shown at the same scale. The figure demonstrates the diversity of spike shapes and sizes for the wheat samples studied.

### 4.3. Simplified Representation of the Spike Contour Model

**Figure 7:**
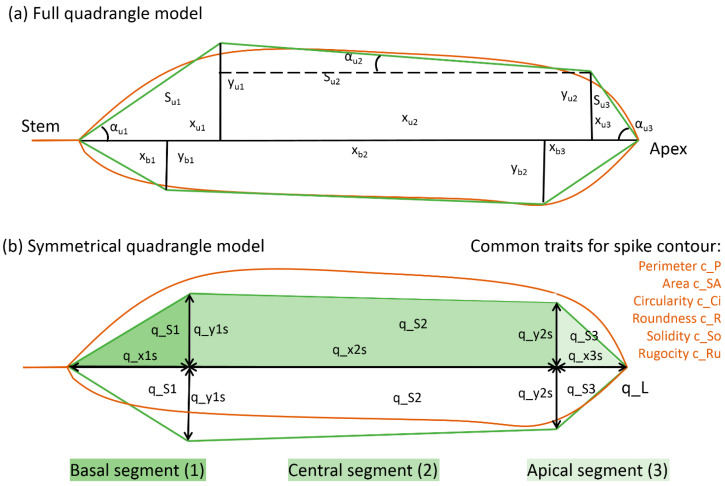
Quadrangle models used to describe the shape and size of the wheat spike. The spike is shown on its side, where its rachis is horizontal with the stem to the left and apex to the right of the figure. The spike body contour curve is shown in brown. Green straight lines show two quadrangles approximating the upper and lower parts of the spike. (**a**) The full model and its parameters described by the WERecognizer program [33]. (**b**) Symmetrical quadrangle model. All parameters for the upper and lower quadrangles are identical. The basal, central and apical segments of the model are filled with different colors.

Using the previously developed phenotyping method [37], the spike images were segmented into the background, spike body and awns. Awns were characterized by their area in the image (c_AA). The WERecognizer program estimated a number of morphometric characteristics of the spike from the image. It used the model of two quadrangles with a common base, which was the spike rachis (Figure 7a). The parameters of the two quadrangles were estimated independently and may have differed. The quadrangle in this model is described by independent parameters: three segment lengths of the quadrilateral base (x_u1_, x_u2_ and x_u3_ for the top quadrangle, where their sum yields the length of the spike) and two heights (y_u1_ and y_u2_). Other parameters (areas of the model segments, angles of inclination of the edges, etc.) could be derived from these parameters.

In this study, the number of parameters of the spike shape model was reduced (Figure 7b). First of all, this model was made symmetric about the spike axis. The parameter values of the upper and lower quadrangles were identical. In this approximation, the parameters were calculated as the average values of the upper and lower quadrangles. For example, q_x1s = (x_u1_ + x_b1_)/2 (Figure 7). This coarsens the description of the spike shape if the spike shape is asymmetric, but it simplifies the data analysis. Second, parameters characterizing the angles of the quadrangle edges in the current model were not used. Thus, for the model presented in Figure 7b, 26 parameters based on image analysis were estimated. These were five independent parameters: the lengths of the basal, central, and apical segments (q_x1s, q_x2s and q_x3s; q_x1s + q_x2s + q_x3s = q_L) and two quadrangle heights (q_y1s and q_y2s) approximating the widths of the basal and apical segments. There were also 15 derived parameters (normalized lengths, segment areas, etc.) and 6 common characteristics of the shape (circularity, perimeter and others; see Figure 7b). All characteristics describing the linear dimensions of the spike were measured in millimeters, and the areas were measured in square millimeters.

In addition, during the annotation of the spike by an expert, the characteristics of its shape and size were measured manually: spike length (SL), front width (SFW), side width (SSW), number of spikelets per spike (SSC) and spike density index (SDI). The spike linear dimensions were measured manually in centimeters and converted to millimeters for the consistency of the analysis. With this in mind, the SDI parameter was calculated by the following formula [10]:

$$SDI=\frac{(SSC-1)*100}{SL},$$

where SL is measured in millimeters.

A complete list of used spike characteristics, their descriptions and units of measurement are summarized in Table 6.

### 4.4. Statistical Analysis

Statistical analysis was performed in the program Past v 4.17 [76]. To visualize the spike model, scripts in the Python language were developed.

## 5. Conclusions

The diversity of spike shapes and sizes was evaluated for seven hexaploid wheat species and one amphidiploid using the geometric quadrangle model. The shape of the wheat spike could be characterized by three main segments—the base, the central part and the apex—using the quadrangle model. Within each of these segments, the parameters exhibited a high degree of statistical dependence. These parameter groups may reflect both the genetic control of the spatial shape and the specific characteristics of the model used to describe it.

The parameters obtained digitally from the wheat spike model had a high correlation with manually measured spike parameters. They allowed for successful differentiation between plant species and spike types using LDA. The results of such analysis showed that spikes from the wheat *T. aestivum* and *T. compactum* species, *T. spelta* species and *T. antiquorum* and *T. sphaerococcum* species were successfully separated by the obtained parameters. The spelt species of *T. spelta* and *Amphiploid speltiforme*, which have a different genetic basis controlling their spelt phenotype, also differed from each other. The model also made it possible to identify spike characteristics differing significantly between species or between accessions within the same species. These results demonstrate the validity of the proposed spike model.

There are, however, several limitations of the current method. It gives systematic bias for some spike parameter estimates, likely due to inherent image analysis errors, it cannot be used directly in spikelet counting or spike density determination, and it does not provide shape analysis for branched spikes.

In the future, the proposed and similar approaches with some improvements can be used for a wide class of tasks related to wheat spike morphometry and the study of genetic control of the spike shape and size.

## Figures and Tables

**Table 1:** Number of spikes analyzed (*n*), mean values and standard errors for the main spike parameters, characterizing its size and shape. The maximum values among all species are shown in bold, and the minimum values are shown in bold and italics.

Trait	ASP	TAE	TAN	TCO	TMA	TSP	TSH	TYU
N	9	50	10	63	9	14	18	8
SL	90.44 ± 6.06	74.64 ± 2.25	39.70 ± 1.35	51.56 ± 0.89	84.22 ± 3.46	**94.71 ± 5.02**	***35.83 ± 1.17***	87.00 ± 3.76
SFW	8.22 ± 0.40	9.12 ± 0.25	8.70 ± 0.15	**11.65 ± 0.24**	***6.33 ± 0.33***	6.46 ± 0.19	8.22 ± 0.32	7.38 ± 0.46
SSW	6.33 ± 0.33	7.52 ± 0.24	6.90 ± 0.18	**8.29 ± 0.20**	6.22 ± 0.36	5.96 ± 0.13	6.61 ± 0.24	7.00 ± 0.46
SSC	**19.22 ± 1.19**	15.60 ± 0.41	17.60 ± 0.37	16.51 ± 0.41	18.78 ± 0.66	15.36 ± 0.39	***14.89 ± 0.27***	17.13 ± 1.06
SDI	20.25 ± 0.71	19.88 ± 0.51	**42.61 ± 2.11**	30.48 ± 0.92	21.29 ± 0.90	**15.58 ± 0.75**	39.30 ± 1.19	18.46 ± 0.55
q_x1s	**31.70 ± 5.46**	30.72 ± 2.07	23.70 ± 5.08	29.60 ± 2.21	22.29 ± 4.70	27.42 ± 4.33	24.31 ± 3.02	***16.27 ± 4.76***
q_x2s	59.96 ± 8.20	45.00 ± 2.99	19.55 ± 2.57	33.07 ± 1.52	55.31 ± 4.03	**65.68 ± 4.11**	***13.60 ± 2.05***	61.96 ± 9.47
q_x3s	9.75 ± 3.85	10.98 ± 1.51	6.36 ± 2.46	6.78 ± 0.61	10.28 ± 2.48	**14.48 ± 3.36**	8.46 ± 1.84	***6.17 ± 2.59***
q_y1s	3.36 ± 0.31	3.71 ± 0.23	3.60 ± 0.51	**4.69 ± 0.26**	3.32 ± 0.29	***2.80 ± 0.55***	3.42 ± 0.61	3.06 ± 0.51
q_y2s	2.81 ± 0.80	3.98 ± 0.22	3.51 ± 0.46	**6.53 ± 0.25**	***2.10 ± 0.18***	2.72 ± 0.63	4.41 ± 0.60	3.17 ± 0.59
q_L	101.41 ± 4.70	86.71 ± 2.58	49.61 ± 4.28	69.46 ± 2.26	87.88 ± 3.32	**107.58 ± 4.62**	***46.37 ± 4.75***	84.40 ± 11.30
q_S1	55.54 ± 9.96	60.98 ± 5.66	45.44 ± 11.10	**78.29 ± 7.19**	37.20 ± 7.11	42.36 ± 7.38	32.58 ± 4.53	***28.74 ± 9.19***
q_S2	163.10 ± 28.25	164.01 ± 11.17	65.08 ± 8.66	178.79 ± 8.70	152.42 ± 19.25	143.95 ± 11.23	***44.34 ± 5.74***	**210.22 ± 50.08**
q_S3	17.33 ± 7.35	**26.92 ± 5.32**	15.65 ± 6.36	25.49 ± 3.22	13.55 ± 3.97	26.38 ± 8.32	19.90 ± 4.97	***12.25 ± 6.02***
q_S	235.97 ± 23.19	251.91 ± 10.84	126.17 ± 10.37	**282.56 ± 9.20**	203.17 ± 19.44	212.69 ± 16.60	***96.82 ± 7.26***	251.22 ± 53.24
q_ym	2.30 ± 0.15	2.89 ± 0.09	2.64 ± 0.21	**4.15 ± 0.12**	2.29 ± 0.18	***1.95 ± 0.09***	2.27 ± 0.18	2.75 ± 0.35
c_P	253.73 ± 15.06	246.19 ± 5.29	***153.73 ± 8.11***	235.90 ± 0.02	207.76 ± 11.16	**274.77 ± 11.41**	176.49 ± 8.16	232.49 ± 19.01
c_SA	489.51 ± 41.04	545.65 ± 20.10	272.73 ± 12.30	**599.75 ± 0.02**	430.03 ± 43.58	453.96 ± 28.00	***261.87 ± 13.62***	558.06 ± 96.85
c_AA	***5.38 ± 1.18***	21.82 ± 1.81	7.18 ± 0.76	**96.73 ± 0.02**	46.64 ± 10.77	57.14 ± 15.20	11.12 ± 1.09	78.24 ± 17.77
c_CI	0.11 ± 0.01	0.15 ± 0.01	0.21 ± 0.03	**0.23 ± 0.01**	0.15 ± 0.01	***0.09 ± 0.01***	0.20 ± 0.03	0.15 ± 0.01
c_R	0.07 ± 0.00	0.10 ± 0.00	0.16 ± 0.03	0.17 ± 0.00	0.07 ± 0.00	***0.06 ± 0.00***	**0.18 ± 0.03**	0.09 ± 0.01
c_So	0.68 ± 0.03	0.71 ± 0.01	0.70 ± 0.03	0.75 ± 0.02	**0.85 ± 0.02**	***0.63 ± 0.03***	0.64 ± 0.03	0.79 ± 0.04
c_Ru	1.18 ± 0.03	1.26 ± 0.03	1.23 ± 0.03	1.25 ± 0.02	***1.07 ± 0.01***	1.26 ± 0.08	**1.29 ± 0.05**	1.21 ± 0.09

**Table 2:** Results of one-factor analysis of variance, Levene’s test and the Kruskal–Wallis test to assess the similarity of spike characteristics in seven wheat species and the amphidiploid. Significant differences (*p* < 0.05) are shown in bold.

Trait	ANOVA		Levene’s Test from Medians	Kruskal–Wallis Test for Equal Medians	
	*F*	*p*	*p*	*Hc* (Tie Corrected)	*p*
SL	63.07	**1.797 × 10^−45^**	**4.961 × 10^−9^**	141.3	**2.66 × 10^−27^**
SFW	9.482	**5.01 × 10^−10^**	**0.0002302**	57.16	**5.554 × 10^−10^**
SSW	27	**3.081 × 10^−25^**	**0.0005318**	103.7	**1.896 × 10^−19^**
SSC	5.649	**0.000006414**	**0.00002497**	39.41	**1.63 × 10^−6^**
SDI	55.53	**5.242 × 10^−42^**	**0.00002992**	138.8	**9.156 × 10^−27^**
q_x1s	1.346	0.2311	0.2123	9.921	0.1931
q_x2s	22.74	**5.17 × 10^−22^**	**0.0001098**	91.23	**6.925 × 10^−17^**
q_x3s	1.851	0.07999	0.05528	9.437	0.2228
q_y1s	3.028	**0.00491**	**0.02729**	24.66	**0.000872**
q_y2s	15.4	**8.629 × 10^−16^**	0.2018	79.47	**1.769 × 10^−14^**
q_L	19.35	**3.015 × 10^−19^**	0.3375	80.42	**1.132 × 10^−14^**
q_S1	3.741	**0.0008219**	**0.001609**	23.95	**0.001161**
q_S2	11.25	**7.933 × 10^−12^**	**0.00008505**	62.04	**5.894 × 10^−11^**
q_S3	0.7227	0.6529	0.3255	8.768	0.2697
q_S	15.95	**2.753 × 10^−16^**	**0.003657**	72.27	**5.138 × 10^−13^**
q_ym	50.81	**1.138 × 10^−39^**	**0.004768**	131	**3.922 × 10^−25^**
c_P	13.39	**6.557 × 10^−14^**	0.8333	62.96	**3.853 × 10^−11^**
c_SA	16.74	**5.364 × 10^−17^**	**0.004782**	74.08	**2.208 × 10^−13^**
c_AA	18.07	**3.666 × 10^−18^**	**3.871 × 10^−16^**	116.1	**4.92 × 10^−22^**
c_CI	10.1	**1.158 × 10^−10^**	**0.01301**	84.56	**1.613 × 10^−15^**
c_R	10.09	**1.192 × 10^−10^**	**0.000001117**	87.43	**4.161 × 10^−16^**
c_So	6.757	**3.934 × 10^−7^**	0.1106	38.11	**0.000002887**
c_Ru	1.457	0.1854	0.3295	27.68	**0.0002516**

**Table 3:** Percentage of correctly predicted spike classes using LDA for different sets of traits and classification classes.

Traits Set/Data Transformation	Percentage of Correctly Classified Spikes	
	Species or Amphidiploid, 8 Classes	Spike Type, 3 Classes
Manually estimated (5 traits)	67.89	88.95
Digitally estimated (26 traits)	78.42	83.16
Combined (31 traits)	87.89	92.11
Manually estimated/Box–Cox	68.42	90.52
Digitally estimated/Box–Cox	82.11	85.26
Combined/Box–Cox	**88.42**	**93.16**

**Table 4:** Results of spike characteristic similarity evaluation in wheat accessions WAG 8326 and k1709 and species *T. compactum*. Number of spikes (*n*), mean value, variance, *p* values for the *t*-test for mean equality, *F*-test for variance equality and Mann–Whitney test results are given. Significant differences (*p* < 0.05) are shown in bold.

	WAG 8326, *n* = 8		k1709, *n* = 9		*t*-Test	*F*-Test	Mann–Whitney
Trait	Mean	Variance	Mean	Variance	*p* (Same Means)	*p* (Same Variances)	*p* (Equal)
SL	43.25	13.36	60.44	13.78	**8.5048 × 10^−8^**	0.98	**0.0006**
SWF	14.00	5.14	12.11	1.11	**0.04**	0.05	**0.02**
SSW	8.25	4.21	8.89	0.86	0.41	**0.04**	0.35
SSC	19.88	7.84	20.44	1.53	0.59	**0.03**	0.92
SDI	43.59	25.59	32.25	5.81	**2.3532 × 10^−5^**	0.05	**0.0006**
q_x1s	15.44	84.86	36.65	433.88	**0.02**	**0.04**	0.06
q_x2s	26.60	79.64	41.15	201.29	**0.02**	0.24	**0.03**
q_x3s	4.65	16.85	6.63	9.47	0.28	0.44	0.16
q_y1s	7.46	3.16	3.24	4.23	**0.0004**	0.71	**0.002**
q_y2s	5.54	5.21	7.34	1.52	0.06	0.11	0.09
q_L	46.69	191.99	84.43	95.06	**9.0908 × 10^−6^**	0.35	**0.0006**
q_S1	56.75	1350.90	79.90	6046.60	0.45	0.06	0.96
q_S2	174.05	4175.40	216.74	4848.20	0.21	0.86	0.27
q_S3	13.62	140.08	26.81	185.19	0.05	0.73	0.05
q_S	244.42	4942.00	323.44	1492.70	**0.01**	0.12	**0.005**
q_ym	5.28	0.65	3.88	0.47	**0.001**	0.65	**0.003**
c_P	252.60	1860.20	262.74	524.53	0.55	0.10	0.74
c_SA	527.99	16,810.00	691.58	4882.80	**0.005**	0.10	**0.003**
c_AA	220.86	2343.60	124.17	2159.90	**0.0008**	0.90	**0.002**
c_Ci	0.33	0.03	0.18	0.00	**0.03**	**1.9949 × 10^−5^**	**0.04**
c_R	0.34	0.02	0.12	0.00	**0.0003**	**0.005**	**0.001**

**Table 5:** Wheat species and the amphidiploid used to analyze the diversity of spike characteristics.

Wheat Species or Amphidiploid	Abbreviation	Number of Accessions	Number of Plants
*T. aestivum* L.	TAE	5	50
*T. spelta* L.	TSP	3	14
*T. antiquorum* Heer ex Udach.	TAN	2	20
*T. compactum* Host	TCO	5	63
*T. macha* Dekapr. et Menabde	TMA	2	9
*T. sphaerococcum* Perc.	TSH	2	18
*T. yunnanense* King	TYU	1	7
*Amphiploid speltiforme*	ASP	1	9

**Table 6:** Description of the spike characteristics used for morphometry. The characteristic abbreviations, names and units are given. See also Figure 7b.

Trait No.	Abbreviation	Name	Measurement Units
Manually estimated parameters			
1	SL	Spike length	mm
2	SFW	Front width	mm
3	SSW	Side width	mm
4	SSC	Spikelet number	dimensionless
5	SDI	Density index	dm^−1^
Independent parameters for the quadrangle model			
6	q_x1s	Length of the basal spike segment	mm
7	q_x2s	Length of the central spike segment	mm
8	q_x3s	Length of the apical spike segment	mm
9	q_y1s	Width of the basal segment	mm
10	q_y2s	Width of the apical segment	mm
Derived parameters for the quadrangle model			
11	q_L	Spike length (q_x1s + q_x2s + q_x3s)	mm
12	q_S1	Area of the basal spike segment	mm^2^
13	q_S2	Area of the central spike segment	mm^2^
14	q_S3	Area of the apical spike segment	mm^2^
15	q_S	Area of the quadrangle for half a spike	mm^2^
16	q_ym	Width index (q_S/q_L)	mm
17	q_x1ns	Normalized length of the basal spike segment (q_x1s/q_L)	dimensionless
18	q_x2ns	Normalized length of the central spike segment (q_x2s/q_L)	dimensionless
19	q_x3ns	Normalized length of the apical spike segment (q_x3s/q_L)	dimensionless
20	q_y1ns	Normalized width of the basal spike segment (q_y1s/q_L)	dimensionless
21	q_y2ns	Normalized width of the apical spike segment (q_y2s/q_L)	dimensionless
22	q_S1S	Normalized area of the basal spike segment (q_S1/q_S)	dimensionless
23	q_S2S	Normalized area of the central spike segment (q_S2/q_S)	dimensionless
24	q_S3S	Normalized area of the apical spike segment (q_S3/q_S)	dimensionless
General size and shape parameters for the spike contour			
25	c_P	Perimeter	mm
26	c_SA	Spike body projection area	mm^2^
27	c_AA	Awn area	mm^2^
28	c_Ci	Circularity	dimensionless
29	c_R	Roundness	dimensionless
30	c_So	Solidity	dimensionless
31	c_Ru	Rugosity	dimensionless

## Data Availability

The original contributions presented in this study are included in the article and Supplementary Materials. The spike image dataset is available at https://zenodo.org/records/13837454, accessed on 27 September 2024.

## Supplementary Materials

The following supporting information can be downloaded at: https://www.mdpi.com/article/10.3390/plants13192736/s1, “Supplementary File S1.xlsx”: Results of the Shapiro–Wilk W test for normality of spike traits in the all-spike sample and individual species. “Supplementary File S2.pdf” contains the following information. Table S1: Results of ANOVA, Levene’s test, and Kruskal–Wallis test to assess the similarity of spike characteristics in three types of spikes. Table S2: Confusion matrix for plant classification by species. Table S3: Confusion matrix for classifying plants by spike type. Table S4: Detailed description of the plant specimen used in the work. Figure S1: Pearson correlation coefficients r for pairs of wheat spike traits. Figure S2: LDA biplot for classification of wheat plants into three spike types.
