# Supplementary material for: Evaluation of the Spike Diversity of Seven Hexaploid Wheat Species and an Artificial Amphidiploid Using a Quadrangle Model Obtained from 2D Images
Source: Plants (Basel). 2024 Sep 30;13(19):2736. doi: 10.3390/plants13192736 (PMC11478967; doi:10.3390/plants13192736)
Supplement: Supplementary file 1 [file plants-13-02736-s001.zip › Supplementary File S2.pdf]

# Supplementary tables and figures for Komyshev et al. paper

**Table S1.** Results of ANOVA, Levene´s test, and Kruskal-Wallis test to assess the similarity of spike characteristics in three types of spikes (compact, normal, and speltoid). Significant differences (*p*<0.05) are marked in bold.

| Trait | ANOVA | | Levene´s test, from medians | Kruskal-Wallis test for equal medians | |
|---|---|---|---|---|---|
| | F | *p* | *p* | Hc (tie corrected): | *p* |
| SL | 182.6 | **8.726E-45** | **0.00001951** | 128 | **1.609E-28** |
| SFW | 17.53 | **1.044E-07** | **0.04675** | 34.14 | **3.865E-08** |
| SSW | 54.76 | **1.816E-19** | **0.000004232** | 79.77 | **4.759E-18** |
| SSC | 4.167 | **0.01695** | 0.7521 | 7.592 | **0.02246** |
| SDI | 126.2 | **1.78E-35** | **1.88E-09** | 121.8 | **3.58E-27** |
| q_x1s | 1.437 | 0.2402 | 0.1207 | 3.155 | 0.2065 |
| q_x2s | 60.34 | **5.70E-21** | **0.004361** | 73.8 | **9.44E-17** |
| q_x3s | 2.75 | 0.06653 | 0.06262 | 3.351 | 0.1872 |
| q_y1s | 5.738 | **0.003813** | **0.002028** | 13.38 | **0.001246** |
| q_y2s | 31.78 | **1.29E-12** | **0.00146** | 55.55 | **8.66E-13** |
| q_L | 45.68 | **6.77E-17** | 0.5884 | 66.52 | **3.58E-15** |
| q_S1 | 4.237 | **0.01585** | **0.01146** | 6.509 | **0.0386** |
| q_S2 | 2.837 | 0.06109 | 0.3941 | 5.129 | 0.07696 |
| q_S3 | 0.9832 | 0.376 | 0.3681 | 3.648 | 0.1614 |
| q_S | 1.473 | 0.2319 | **0.03268** | 3.402 | 0.1825 |
| q_ym | 34.46 | **1.78E-13** | **1.46E-09** | 52.85 | **3.34E-12** |
| c_P | 11.32 | **2.28E-05** | 0.3071 | 21.14 | **2.57E-05** |
| c_SA | 2.12 | 0.1229 | **0.03512** | 4.895 | 0.0865 |
| c_AA | 9.621 | **0.0001052** | **1.80E-06** | 6.9 | **0.03175** |
| c_CI | 29.4 | **7.77E-12** | **0.0006935** | 63.57 | **1.57E-14** |
| c_R | 33.29 | **4.19E-13** | **2.44E-08** | 83.08 | **9.12E-19** |
| c_So | 0.2249 | 0.7988 | 0.2709 | 0.2992 | 0.861 |
| c_Ru | 1.756 | 0.1756 | 0.6349 | 15.74 | **0.0003815** |

**Table S2.** Confusion matrix for plant classification by species (full feature set, Box-Cox transformation applied to input data). Erroneous classifications are shown in red. Number of correct predictions 168.

| | ASP | TAE | TAN | TCO | TMA | | TSP | TSH | TYU | Total |
|---|---|---|---|---|---|---|---|---|---|---|
| ASP | 8 | 1 | 0 | 0 | 0 | | 0 | 0 | 0 | 9 |
| TAE | 4 | 39 | 0 | 2 | 3 | | 0 | 0 | 2 | 50 |
| TAN | 0 | 0 | 18 | 0 | 0 | | 0 | 2 | 0 | 20 |
| TCO | 0 | 0 | 0 | 63 | 0 | | 0 | 0 | 0 | 63 |
| TMA | 0 | 0 | 0 | 0 | 8 | | 0 | 0 | 1 | 9 |
| TSP | 4 | 0 | 0 | 0 | 0 | | 10 | 0 | 0 | 14 |
| TSH | 0 | 0 | 2 | 0 | 0 | | 0 | 16 | 0 | 18 |
| TYU | 1 | 0 | 0 | 0 | 0 | | 0 | 0 | 6 | 7 |
| Total | 17 | 40 | 20 | 65 | 11 | | 10 | 18 | 9 | 190 |

**Table S3**. Confusion matrix for classifying plants by spike type (full feature set, Box-Cox transformation applied to input data). Erroneous classifications are shown in red. Number of correct predictions 177.

| | Spelt | Normal | Compact | Total |
|---|---|---|---|---|
| Spelt | 34 | 5 | 0 | 39 |
| Normal | 6 | 43 | 1 | 50 |
| Compact | 0 | 1 | 100 | 101 |
| Total | 40 | 49 | 101 | 190 |

**Table S4.** Detailed description of the plant specimen used in the work.

| Species | Accession/Cultivar | Country of origin | Reproduction date, month-year | Plants= images |
|---|---|---|---|---|
| *T. aestivum* | k1386/#37* | Tajikistan | IX-2016, V-2017 | 18 |
| | AUS 90069/Triple Dirk D | Australia | IX-2016 | 1 |
| | ANK-23 | Russia | IX-2016, II-2018 | 17 |
| | Babilo | Tajikistan | IX-2016 | 10 |
| | Novosibirskaya 67 | Russia | II-2017 | 4 |
| *T. spelta* | K-53660** | Tajikistan | IX-2016 | 1 |
| | K-19092** | Latvia | II-2018 | 7 |
| | K-1731/ Rother Sommer Kolben** | Germany | IX-2016, II-2018 | 6 |
| *T. antiquorum* | k-56397 | Tajikistan | IX-2016 | 10 |
| | k-56398 | Tajikistan | IX-2016 | 10 |
| *T. compactum* | WAG 1326*** | Netherlands | IX-2016 | 1 |
| | WAG 8326*** | Netherlands | IX-2016 | 8 |
| | k1709/#29* | Tajikistan | IX-2016, V-2016 | 9 |
| | k1711/#31* | Tajikistan | IX-2016, V-2016 | 18 |
| | k1713/#33* | Tajikistan | V-2016 | 27 |
| *T. macha* | k-28195 | Georgia | II-2018 | 6 |
| | k-28170 | Georgia | II-2018 | 3 |
| *T. sphaerococcum* | k-14976* | Pakistan | II-2018 | 8 |
| | k-33750* | Pakistan | II-2018 | 10 |
| *T. yunnanense* | KU 508**** | China | IX-2016 | 7 |
| *Amphyploid speltiforme* | Aminov | Azerbaijan | V-2016 | 9 |

* - H.Yusufbekov Pamir Biological Institute, National Academy of Sciences of Tajikistan (Khorog, Tajikistan); ** - VIR; *** - Wageningen Agricultural University (the Nederland); **** - Kyoto Univ.; ***** - Genetic Resources Institute of the Azerbaijan National Academy of Sciences, Baku, Azerbaijan; others – collection by the Wheat genetics sector of the IC&G SB RAS, Novosibirsk, Russia.

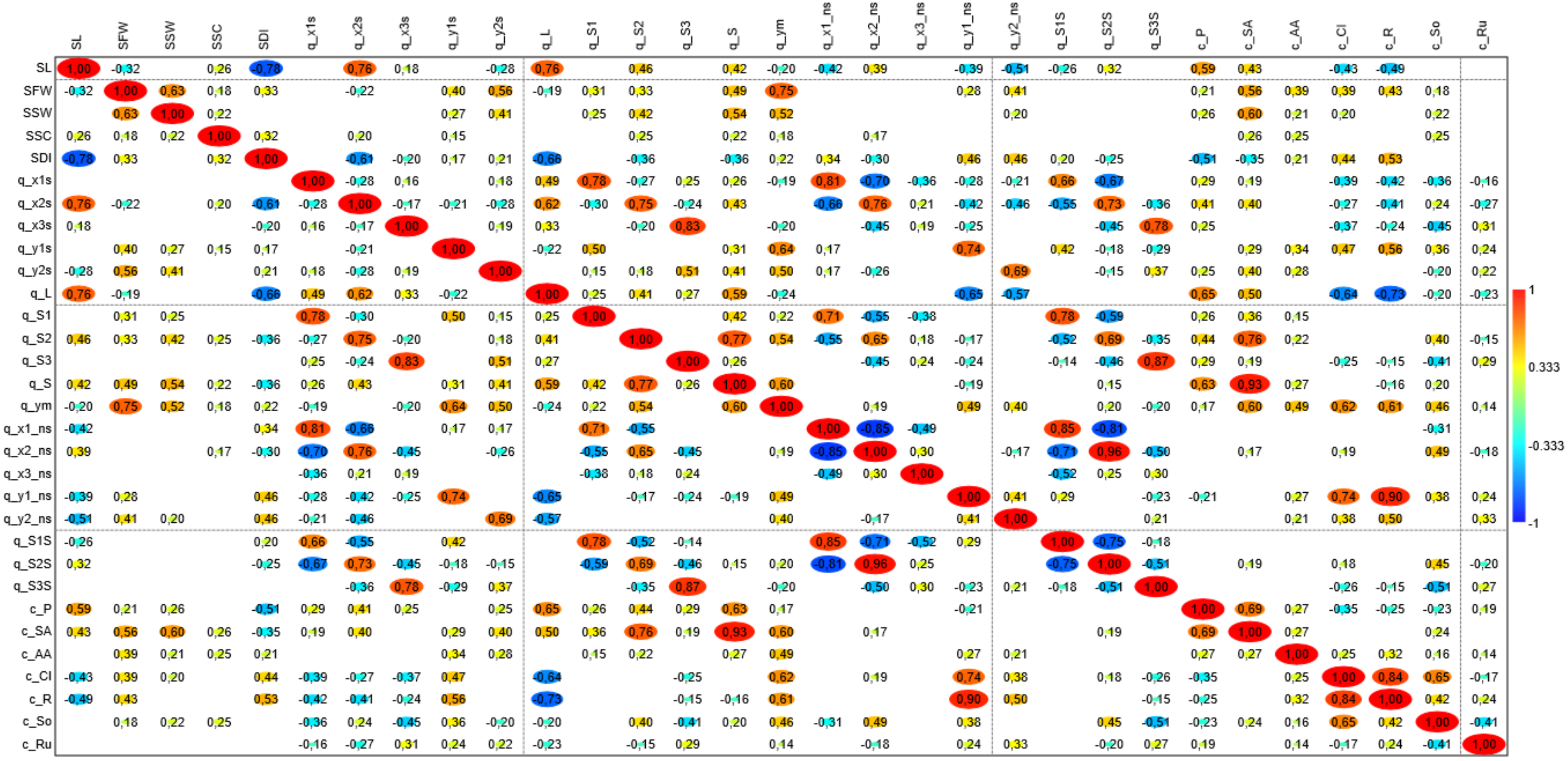


**Figure S1.** Pearson correlation coefficients *r* for pairs of wheat spike traits. 5 traits estimated manually and 26 traits obtained from image analysis are separated by solid dark grey line. Non-significant *r* values ($p>0.05$) are not shown in the figure. The correspondence between *r* and color is shown to the right of the figure.

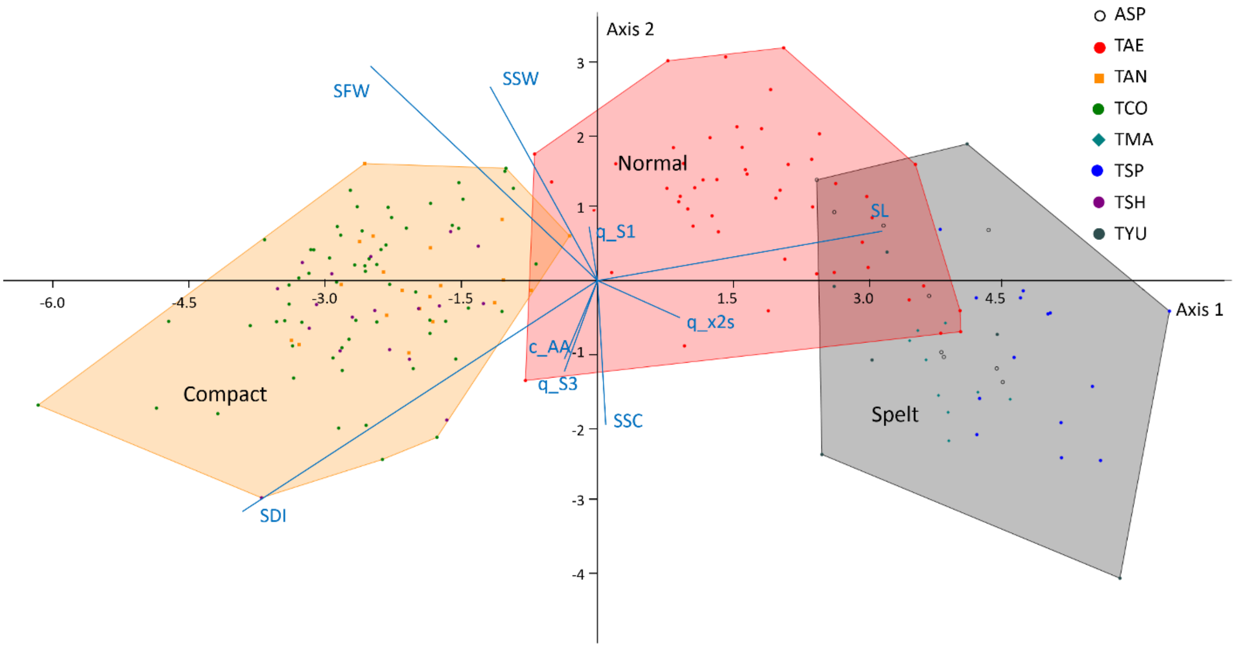


**Figure S2.** LDA biplot for classification of wheat plants into three spike types using combined set of species and Box-Cox transformation for input data. Polygons for 3 classes are shown by different color. The marker types for species/hybrid are shown to the right of the diagram. Projections for most important traits are shown by blue lines.
